# A Combined Measure of Procedural Volume and Outcome to Assess Hospital Quality of Colorectal Cancer Surgery, a Secondary Analysis of Clinical Audit Data

**DOI:** 10.1371/journal.pone.0088737

**Published:** 2014-02-18

**Authors:** Nikki E. Kolfschoten, Perla J. Marang-van de Mheen, Michel W. J. M. Wouters, Eric-Hans Eddes, Rob A. E. M. Tollenaar, Theo Stijnen, Job Kievit, on behalf of the Dutch Surgical Colorectal Audit Group

**Affiliations:** 1 Department of Surgery, Leiden University Medical Centre, Leiden, the Netherlands; 2 Department of Medical Decision Making, Leiden University Medical Centre, Leiden, The Netherlands; 3 Department of Surgical Oncology, Netherlands Cancer Institute, Antoni van Leeuwenhoek hospital Amsterdam, Amsterdam, The Netherlands; 4 Department of Surgery, Deventer Hospital, Deventer, The Netherlands; 5 Department of Biostatistics, Leiden University Medical Centre, Leiden, The Netherlands; University Hospital Heidelberg, Germany

## Abstract

**Objective:**

To identify, on the basis of past performance, those hospitals that demonstrate good outcomes in sufficient numbers to make it likely that they will provide adequate quality of care in the future, using a combined measure of volume and outcome (CM-V&O). To compare this CM-V&O with measures using outcome-only (O-O) or volume-only (V-O), and verify 2010-quality of care assessment on 2011 data.

**Design:**

Secondary analysis of clinical audit data.

**Setting:**

The Dutch Surgical Colorectal Audit database of 2010 and 2011, the Netherlands.

**Participants:**

8911 patients (test population, treated in 2010) and 9212 patients (verification population, treated in 2011) who underwent a resection of primary colorectal cancer in 89 Dutch hospitals.

**Main Outcome Measures:**

Outcome was measured by Observed/Expected (O/E) postoperative mortality and morbidity. CM-V&O states 2 criteria; 1) outcome is not significantly worse than average, and 2) outcome is significantly better than substandard, with ‘substandard care’ being defined as an unacceptably high O/E threshold for mortality and/or morbidity (which we set at 2 and 1.5 respectively).

**Results:**

Average mortality and morbidity in 2010 were 4.1 and 24.3% respectively. 84 (94%) hospitals performed ‘not worse than average’ for mortality, but only 21 (24%) of those were able to prove they were also ‘better than substandard’ (O/E<2). For morbidity, 42 hospitals (47%) met the CM-V&O. Morbidity in 2011 was significantly lower in these hospitals (19.8 vs. 22.8% p<0.01). No relationship was found between hospitals' 2010 performance on O-O en V-O, and the quality of their care in 2011.

**Conclusion:**

CM-V&O for morbidity can be used to identify hospitals that provide adequate quality and is associated with better outcomes in the subsequent year.

## Introduction

Increasingly, society demands that health care providers demonstrate that the quality of the care they provide is adequate. However, it is not clear how quality should be measured and judged. Quality of health care has been defined as “the degree to which health services for individuals and populations increase the likelihood of desired health outcomes and are consistent with current professional knowledge”[1]. Patient safety (the prevention of unintended harm) is an essential element of quality of care.

Assessing quality, in particular patient safety, on the basis of outcome, i.e. the occurrence of adverse outcomes, has proven unreliable. The incidence of adverse outcomes is usually low, so the absence of adverse outcomes in a small series of patients is likely even if care were substandard. In the same way, when procedural volume is low, even an adverse event rate of 3 or 5 times average may still be ‘not significantly worse than average’[2].

For this reason, and because higher procedural volume is associated with better outcome, the emphasis in quality assessment has shifted from outcome to volume. Procedural volume has thus become a surrogate measure for quality [3], [4]. Accordingly, political focus in the Netherlands now aims at concentrating care into high volume centres. Recently, the Association of Surgeons of the Netherlands (ASN) has set the minimal annual procedural volume for colorectal resections at 50 procedures per hospital per year. However, any volume criterion is arbitrary and ignores the fact that lower volumes (e.g. 45 per year) do not exclude high quality, just as high volumes do not rule out substandard care. Therefore assessing quality on the basis of volume only, ignoring outcome, is as inadequate as assessing it by outcome only, ignoring volume. We therefore propose a quality measure that combines volume and outcome, and corrects for case-mix variation to provide statistical evidence that care is both ‘not significantly worse than average’ as well as ‘significantly better than substandard’. A hospital that meets both criteria deserves public confidence that its quality of care is adequate.

**The aim** of the present study is to elucidate and test the proposed method and compare hospitals by three measures to define adequate quality: 1) outcome only (O-O), 2) volume only (V-O), and 3) a combined measure of volume and outcome (CM-V&O). We aim to demonstrate that the CM-V&O not only has a better theoretical basis, but that it also identifies hospitals with better outcome in the subsequent year.

## Patients and Methods

### Patients

We used the database of the Dutch Surgical Colorectal Audit (DSCA, www.DSCA.nl), which has been created in accordance with principles pioneered earlier in the UK and the Scandinavian countries[5]. Details on procedures, data registration and data validity have been described in a recent paper[6]. For the present study (for which no ethical approval was required) we used data from patients treated in 2010 as study database, and validated our measures on patients treated in 2011. The study population consisted of 8,911 patients who underwent a resection for a primary colorectal cancer during 2010 in one of 89 participating Dutch hospitals. The verification population consisted of 9,212 patients, treated in the same 89 hospitals in 2011. These databases include 93% of all patients treated, and 96% of Dutch hospitals. An observation period of 1 year was chosen, as this is the time-span commonly used for benchmarking in quality assurance. Data included 15 case-mix factors (age, gender, Body Mass Index (BMI), preoperative ASA-score, Charlson comorbidity-score[7], history of previous abdominal surgery, Tumour Node Metastasis (TNM) stage, preoperative radiation therapy, preoperative tumour complications (perforation, obstruction or other), multiple synchronous tumors, urgency and type of procedure (right, left/transverse, sigmoid, low anterior or abdomino-perineal resection, and/or extended resection for locally advanced tumour or metastases), as well as outcome. Outcome was assessed by postoperative mortality, occurring either during hospital admission or within 30 days after resection, and/or by serious morbidity, i.e. leading to an intervention (operative or percutaneous) or to prolonged hospital stay (14 days or more).

### Analyses

Risk-adjusted Observed/Expected (O/E) outcome ratio was used as the basic measure of hospital specific quality of care.[8], [9] Observed outcome is the number of adverse outcomes (mortality or morbidity) that occur in a particular hospital in one year, while expected outcome is the sum of all patients' estimated probabilities for these outcomes in that same hospital that year. These probability estimates for patients' mortality and morbidity were derived from a backwards-stepwise multivariate logistic regression model, fitted on the data of that year, of all hospitals. For each of the 89 hospitals studied, O/E outcome ratios (including the exact Poisson 95% confidence intervals) were calculated both for mortality and morbidity, and for 2010 and 2011 separately[10]. For a hospital with average performance, the observed outcome will equal the expected outcome, resulting in an O/E outcome ratio of 1. Hospitals that perform better than average have an O/E outcome ratio lower than 1, while this ratio is higher than 1 in hospitals with poorer than average performance.

We compared the 89 hospitals according to 3 different quality measures:

- **Outcome-only**, this (historical) measure assesses whether patient outcomes are not worse than a predefined ‘threshold for substandard care’. For the present study, the base-case threshold for substandard care was defined as an O/E outcome ratio of 2 for mortality, and of 1.5 for morbidity (and was varied in sensitivity-analyses to 1.5 and 3). Note that this approach ignores the existence and size of a confidence interval (which depends partly on volume) around the point estimate of the O/E ratio.
- **Volume-only**, a more recently proposed measure, assesses whether the volume of procedures (irrespective of outcome) is at least as high as the (arbitrary) threshold of 50 colorectal resections per year set by the Association of Surgeons of the Netherlands (including those for benign diseases). As we did not have any information on benign procedures, for the present study the volume threshold was defined as at least 50 colorectal cancer resections in the year 2010.
- A **combined measure of volume and outcome**, assesses not only whether outcome is adequate, but in addition whether patient volume is sufficiently high to narrow the uncertainty around the observed outcome to an acceptable range. In the CM-V&O, the minimal volume is therefore not a normative threshold, but a statistical condition for reliable assessment of hospital outcome. To pass this measure, a hospital should meet two criteria:
  1. its O/E outcome ratio must be ‘not significantly worse than average’, i.e. the lower limit of the 95% confidence interval (CI95min for short) of its O/E outcome ratio should be no higher than 1, and
  2. its O/E outcome ratio must be ‘significantly better than substandard’, i.e. the upper limit of the 95% confidence interval (CI95max for short) of its O/E outcome ratio should be lower than the predefined threshold for substandard care (see A).

Conceptually, these two criteria mean ‘no proof of care being bad’ and ‘sufficient proof of care being OK’ respectively, with the burden of proof lying in particular with the hospital for the second criterion.

### Verification

A good measure is not only *discriminative*, meaning that it will identify the adequately performing hospitals and detect the hospitals with insufficient quality, but also *reliable*, meaning that it will not only do so for the year measured, but also the following year. To validate the reliability of the CM-V&O, we compared 2011 outcomes between hospitals meeting or failing different measures in 2010, to see if hospitals' quality of care in 2011 was predicted by their performance on the measures O-O, V-O and CM-V&O in 2011. To translate the O/E outcomes to clinical outcomes, we also calculated the risk-adjusted outcomes (O/E outcome multiplied by the national average outcome). Also, we also performed the reversed analysis: we investigated if the different measures also detected hospitals with insufficient quality the following year by calculating how many of the hospitals that were ‘significantly worse than average’ in 2011, were detected by the different quality measures as delivering substandard care in 2010 (i.e. NOT meeting the different measures).

All statistics were performed in PASW Statistics for Mac, Rel 18.0.2009. Chicago: SPSS inc. and Microsoft Excel.

## Results

### Patients

**Figure 1:**
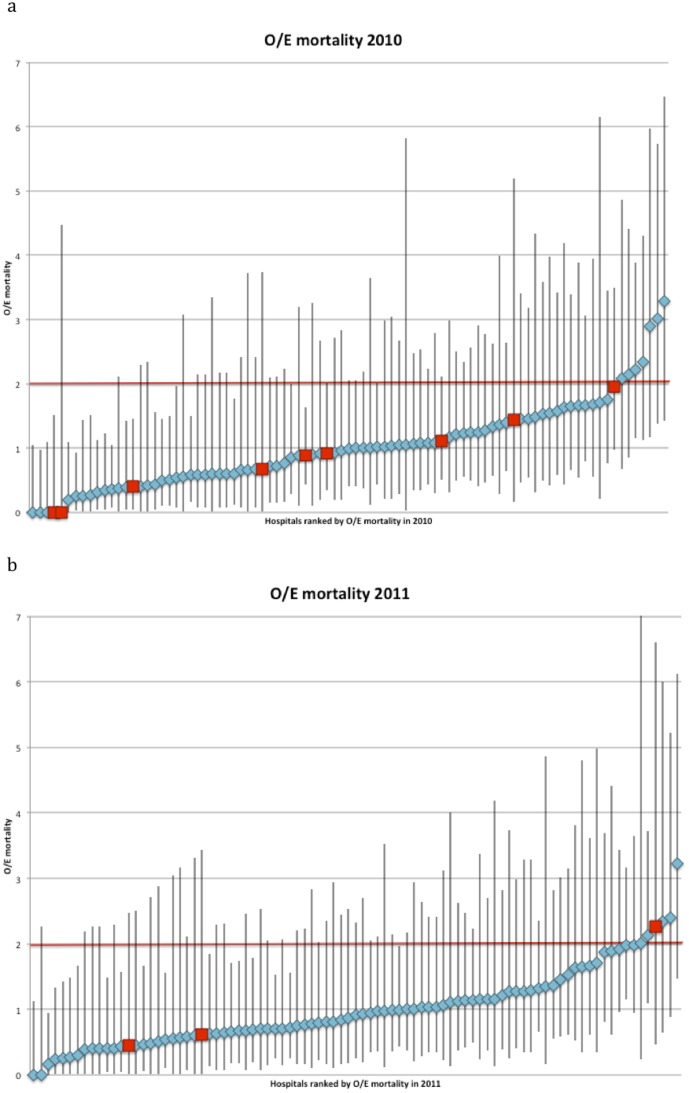
Observed/Expected (O/E) mortality ratio of all hospitals in the Dutch Surgical Colorectal Audit in 2010 and 2011. *Measure of ‘Outcome only’*: requires that a hospital's O/E ratio, regardless of its confidence interval, is below or equal to 2 (red fat printed line), met the ‘outcome only’ criterion. *Measure of ‘Volume only’*: requires that a hospital meets the ‘volume only’ criterion of >50 procedures per year (red squares) *Combined measure of ‘volume and outcome (CM-V&O)’*: the lower limit of the confidence interval around the hospitals O/E ratio is below or equal to 1, i.e. that the hospital is not significantly worse than average. In addition CM-V&O requires that the higher limit of the confidence interval is below 2 (fat printed red line), i.e. that the hospital is significantly better than substandard.

**Figure 2:**
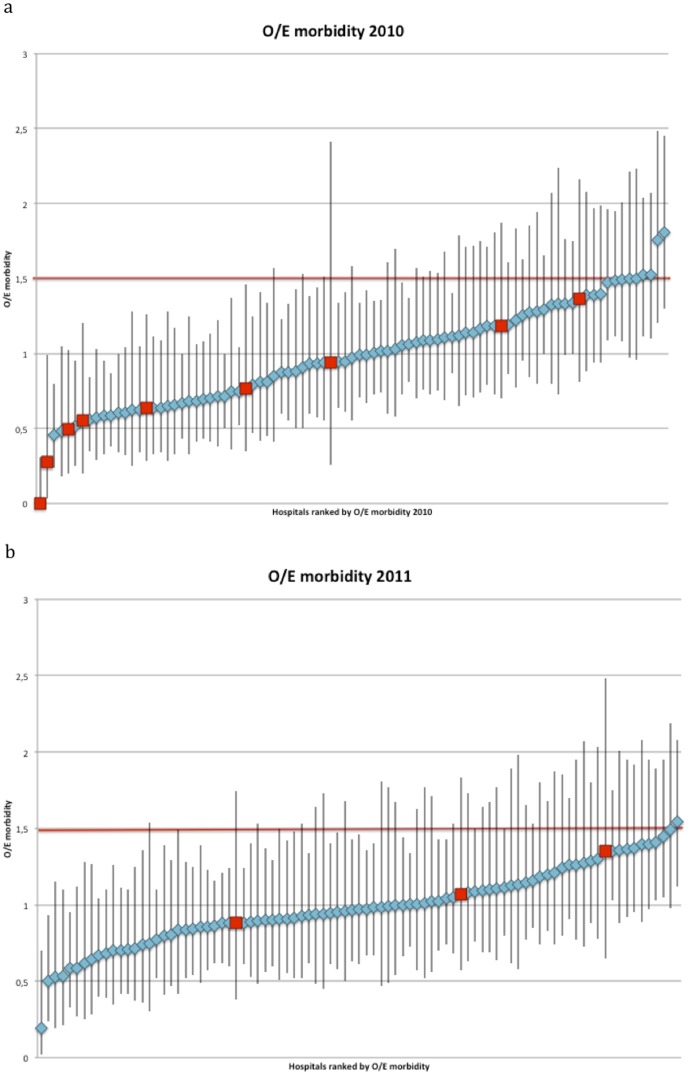
Observed/Expected (O/E) morbidity ratio of all hospitals in the Dutch Surgical Colorectal Audit in 2010 and 2011. *Measure of ‘Outcome only’*: requires that a hospital's O/E ratio, regardless of its confidence interval, is below or equal to 1.5 (red fat printed line), met the ‘outcome only’ criterion. *Measure of ‘Volume only’*: requires that a hospital meets the ‘volume only’ criterion of >50 procedures per year (red squares) *Combined measure of ‘volume and outcome (CM-V&O)’*: requires that the lower limit of the confidence interval around the hospitals O/E ratio is below or equal to 1, i.e. that the hospital is not significantly worse than average. In addition CM-V&O requires that the higher limit of the confidence interval is below 1.5 (fat printed red line), i.e. that the hospital is significantly better than substandard.

The study population (2010) consisted of 8,911 patients treated in 89 hospitals, with an average procedural volume of 100 patients per year (range 14–241). The population average mortality and morbidity rates were 4.1% and 24.3% respectively. Seven out of 15 case-mix factors (age, gender, ASA, Carlson score, type of procedure, preoperative radiation therapy and urgency) predicted mortality with good accuracy. Morbidity was predicted by 12 factors (all except BMI, TNM-stage and synchronous metastasectomy). The model predicting mortality, and that predicting morbidity, had similar test-characteristics (C-statistics 0.80 CI95 0.78–0,82 and 0.75 CI95 0.72–0.78 respectively). The two models were used to calculate expected mortality and expected morbidity, and O/E mortality and morbidity ratios for each hospital. The average hospital O/E mortality ratio was 1.04 (CI95 0.90–1.19), the average O/E morbidity ratio 0.97 (CI95 0.90–1.04). (Figure 1 and 2).

The verification population (2011) consisted of 9,212 patients, treated in the same 89 hospitals. Average morbidity and mortality rates decreased significantly in 2011, as compared to 2010: 3.7% (p<0.01) and 21.5% (p<0.01) respectively, as described previously.[11] Table 1 shows patient, tumour and treatment characteristics and outcome in the DSCA in the study population of 2010 and the verification population of 2011.

**Table 1:** Patient, tumour and treatment characteristics and outcome in the DSCA 2010 and 2011.

Case-mix factors		2010	2011
		N (%)	N (%)
Age (years)	<70	4006 (45)	4222 (46)
	70–79	2924 (33)	3038 (33)
	80+	1975 (22)	1935 (21)
Gender	Male	4914 (55)	5065 (55)
Previous abdominal surgery	Yes	2925 (33)	3087 (34)
	Missing	132 (1.5)	117 (1.3)
ASA	III+	2094 (24)	2156 (23)
	Missing	82 (1)	60 (1)
Charlson	2+	969 (11)	1054 (11)
BMI	<25	3248 (36)	3678 (40)
	25–30	2769 (31)	3468 (38)
	>30	1169 (13)	1362 (15)
	Missing	1725 (19)	703 (8)
TNM stage	I and II	4884 (55)	4900 (53)
	III	2769 (31)	2896 (31)
	IV	1053 (12)	1053 (11)
	X	205 (2)	363 (4)
Synchronous tumours	Yes	246 (3)	311 (3)
Neoadjuvant therapy	Radiotherapy 5×5 gy	1257 (14)	1268 (14)
	Radiotherapy >60 gy	173 (2)	256 (3)
	Chemo-radiation	727 (8)	789 (9)
Preoperative complication	Feacal peritonitis	153 (2)	117 (1)
	Obstruction	798 (9)	103 (11)
	Other		
Urgency	Urgent	794 (9)	679 (7)
	Acute	487 (6)	648 (7)
Procedure	Right hemi-colectomy	3142 (35)	3013 (33)
	Left hemi-colectomy*	830 (9)	892 (10)
	Sigmoid/LAR	3783 (43)	4107 (45)
	Abdomino-perineal resection	732 (8)	812 (9)
	Other ∧	442 (5)	388 (4)
Additional resection	Locally advanced tumour	840 (9)	875 (10)
	Metastasis	250 (3)	351 (4)
			
Mortality		365 (4.1)	343 (3.7)
Morbidity		2167 (24.3)	1982 (21.5)

ASA =  American Society of Anaesthesiologists classification BMI =  Body Mass Index, TNM =  Tumour Node Metastasis classification system, gy =  gray LAR =  Low anterior resection, *  =  including transverse colectomies, ∧  =  including subtotal or proctocolectectomy.

### Three measures for adequate quality in 2010

Using **O-O** as the measure for adequate quality, 82 (92%) of the hospitals met the O/E ratio ≤2 for mortality, and 84 (94%; see table 2) met the O/E ratio ≤1.5 for morbidity.

**Table 2:** Number of hospitals meeting the measure of ‘outcome-only’ (better than substandard), of ‘volume-only’ (50 procedures or more), or the combined measure of volume and outcome’ in 2010, using different thresholds for ‘substandard care’.

Quality measure		Outcome only	Volume only	Combined measure of volume and outcome		
Outcome measure	Threshold for substandard care	O/E < = substandard (%)	N > = 50 (%)	Not worse than average: CI95min < = 1 (%)	Better than substandard: CI95max < threshold (%)	Both (%)
	O/E<1.5	71 (80)			13 (15)	13 (15)
Mortality	O/E<2	82 (92)	80 (90)	84 (94)	21 (24)	21 (24)
	O/E<3	87 (98)			59 (66)	56 (63)
	O/E<1.5	84 (94)			47 (53)	42 (47)
Morbidity	O/E<2	89 (100)	80 (90)	81 (90)	77 (87)	74 (83)
	O/E<3	89 (100)			89 (100)	81 (90)

N =  number of patients.

O/E =  Observed/Expected outcome ratio.

CI95min  =  Lower limit of 95% confidence interval around the O/E ratio.

CI95max  =  Upper limit of 95% confidence interval around the O/E ratio.

With respect to **V-O**, 9 hospitals (10%) performed less than 50 resections for primary colorectal cancer, while the remaining 80 hospitals (90%) met the volume threshold of 50 or more (table 2).

**CM-V&O** states, as explained before, 2 criteria:

- Criterion 1): being ‘not significantly worse than average’

For mortality, 84 hospitals (94%) had an O/E mortality ratio which was ‘not significantly worse than average’, CI95min < = 1 (table 2). Three of these hospitals even performed significantly better than average (i.e. even CI95max <1). The remaining 5 hospitals had an O/E mortality ratio worse than average (i.e. CI95min >1). For morbidity, 81 (90%) hospitals had an O/E morbidity ratio that was ‘not significantly worse than average’ (table 2), of which 11 hospitals were even significantly better than average. In the remaining 8 hospitals, O/E morbidity ratio was significantly worse than average. One hospital performed significantly worse than average on both mortality and morbidity, and one hospital was significantly better than average on both outcome measures. None of the hospitals that were significantly better than average on one outcome measure were ‘significantly worse than average’ on the other.

- Criterion 2): being ‘significantly better than substandard’

At an O/E outcome ratio of 2 as the threshold for substandard care, 21 hospitals (24%) were ‘significantly better than substandard’ (i.e. CI95max <2, see table 2). All of these hospitals were also ‘not significantly worse than average’ and thus met both criteria of CM-V&O. For morbidity, 77 hospitals were ‘significantly better than substandard’ (CI95max <2), of which 74 met both criteria. All hospitals that met both criteria for mortality also did so for morbidity.

### Using different thresholds for ‘substandard care’

If we had used a stricter threshold for substandard care, such as an O/E outcome ratio of 1.5, the number of hospitals meeting CM-V&O would have dropped to just 13 for mortality, and to 42 for morbidity (table 2). Using a more lenient threshold for substandard care of 3, 56 hospitals would have met CM-V&O for mortality, and 81 for morbidity (table 2).

### Comparing the three quality measures

Table 3 shows that 61 hospitals met the O-O measure for mortality, but had insufficient procedural volume to assess hospital postoperative outcome reliably. As a result of this their CI95max ranged up to 7 times the expected mortality. For morbidity, 37 hospitals met the O-O measure, but not the CM-V&O. Similarly, 61 hospitals met the V-O measure for mortality but outcomes were inadequate to meet the CM-V&O (for morbidity: 39 hospitals). Among these 61 hospitals there were also the 5 hospitals with an O/E mortality ratio significantly worse than average (for morbidity: 8 hospitals). On the other hand, there were also 2 hospitals that did meet the CM-V&O, but not the V-O measure (for morbidity: 6 hospitals).

**Table 3:** Comparing the number of hospitals meeting (or failing) the 3 different measures.

Quality measures met in 2010			Combined measure of volume and outcome 2010*)	
			yes	No
	outcome only*	O/E = <2	21	61
Mortality		O/E>2	0	7
	volume only	N = >50	19	61
		N<50	2	7
	outcome only*	O/E = <1.5	47	37
Morbidity		O/E>1.5	0	5
	volume only	N = >50	41	39
		N<50	6	3

O/E =  Observed/Expected outcome ratio.

N =  Hospital procedural volume.

*) using O/E ratio of >2 as the threshold for substandard care for mortality, and O/E ratio of >1.5 as the threshold for substandard care for morbidity.

### Verification

Hospitals meeting the CM-V&O for mortality in 2010 had a lower risk-adjusted mortality, than hospitals that did not meet the CM-V&O in 2010, but the difference was not statistically significant (3.3 vs 3.9%, n.s.) [Table 4]. Hospitals that met the V-O measure in 2010 had a higher, rather than lower, risk-adjusted mortality in 2011. The CM-V&O detected 2 of 3 hospitals which performed significantly worse than average in 2011 (these hospitals did not meet the CM-V&O in 2010), while none of the other measures detected any of the significantly worse than average hospitals in 2011 (data not shown). For morbidity, hospitals meeting the CM-V&O in 2010 had a significantly lower risk-adjusted morbidity in 2011 (19.8 vs 22.8%, p<0.05), [Table 4] while this effect was not found for the O-O or V-O measure. The CM-V&O detected 3 out of 4 hospitals that were significantly worse than average in 2011, while the O-O measure detected only one, and the V-O measure detected none of the significantly worse than average hospitals in 2011 (data not shown).

**Table 4:** Verification: Outcomes in 2011 of hospitals meeting the different measures in 2010.

**Mortality 2010**		O/E Mortality 2011 (CI95%)	Mortality 2011 (riskadjusted)
Outcome <2 2010	Yes (82)	0.98 (0.88–1.09)	3.6%
	No (7)	1.36 (0.93–1.98)	5.0%
Volume >50 in 2010	Yes (80)	1.01 (0.90–1.12)	3.7%
	No (9)	0.89 (0.59–1.35)	3.3%
CM-V&O 2 2010	Yes (21)	0.89 (0.72–1.10)	3.3%
	No (68)	1.04 (0.92–1.18)	3.9%
CM-V&O 3 2010	Yes (56)	0.98 (0.80–1.19)	3.6%
	No (33)	1.01 (0.89–1.15)	3.7%
Morbidity 2010		O/E Morbidity 2011 (CI95%)	Morbidity 2011 (riskadjusted)
Outcome <1.5	Yes (84)	0.99 (0.95–1.04)	21.3%
	No (5)	1.18 (0.99–1.41)	25.5%
Volume >50	Yes (80)	1.00 (0.96–1.05)	21.6%
	No (9)	0.94 (0.78–1.14)	20.3%
**CM-V&O 1.5** *	**Yes (47)**	**0.92 (0.89–1.00)**	**19.8%**
	**No (42)**	**1.06 (1.01–1.14)**	**22.8%**
**CM-V&O 2** *	**Yes (74)**	**0.97 (0.92–1.02)**	**20.8%**
	**No (15)**	**1.16 (1.05–1.28)**	**25.0%**

CM-V&O =  combined measure for volume and outcome.

O/E =  Observed/Expected outcome ratio.

CI95% = 95% confidence interval around the O/E ratio.

* P<0.05.

## Discussion

In the present study we propose and test a (risk-adjusted) combined measure of volume and outcome to assess the quality of care provided by hospitals. Hospitals that meet this quality measure have not only demonstrably good health care outcomes, but had sufficient annual numbers to demonstrate that their results are not just a ‘lucky streak’, but a manifestation of consistently good underlying quality of care. We demonstrated in the verification population that hospitals meeting the CM-V&O for morbidity had a significantly lower morbidity in the following year. A similar trend was found for mortality; however this did not reach statistical significance. Both the ‘volume only’ and ‘outcome only’ measure did not identify the hospitals with better outcomes the following year. Also, the CM-V&O performed better in detecting hospitals that performed significantly worse than average the next year.

The advantage of our study is that all analyses were performed on large, reliable databases, containing almost nine thousand patients per year from 89 hospitals. However, this also illustrates that such calculations can only be performed if outcome registration is excellent and relevant case-mix factors are included. Fortunately, increasing awareness of the need for quality assurance has led to an increasing dedication to reliable outcome registration by means of National Clinical Audits. In the Netherlands, the first Clinical Audit, the DSCA was initiated in 2009. Although participation, completeness and validity were overwhelmingly good after only one year of registration, the availability of weekly online feedback data on hospital performances was relatively new in the two years described in this study. In three years after the introduction of clinical auditing, a significant improvement in various process and outcome measures was observed, while variation in hospital performances decreased. [11] These improvements may have interfered with the results of our study as hospitals with substandard outcomes may have felt a greater incentive for improvement of these outcomes, reducing the possible differences in outcome in 2011 between hospitals that did and did not meet the different measures. Possibly, when this selective quality improvement would not have taken place, hospitals meeting the CM-V&O in 2010 would also have had a lower risk adjusted mortality rate in 2011.

The ultimate measure for hospital performances on outcomes of care would be *discriminative* and *reliable*, but also understandable for all stakeholders. Although previous studies have found that a high procedural volume for colorectal cancer is associated with better outcome,[12] we found that the recently implemented minimal volume chosen by the ASN (the ‘volume only’ measure) was not *discriminative*, as it excluded hospitals with reliable and good outcomes, and included hospitals with significantly worse than average outcomes. We found that the measure was not *reliable*, as hospitals with insufficient volume according to the ASN, although few, did not perform worse than the hospitals with sufficient numbers in 2011, but that their outcomes, although not significant, were even better. Possibly, a cut-off point of 50 procedures is too low to identify high-quality hospitals. However, our results are in line with recent evidence that suggests that centralization only results in an improvement of outcome if patients are referred to hospitals with a better outcome: outcome-based referral[13]–[16]. On the other hand, we have also shown that selecting hospitals based on ‘outcome only’, was also not *reliable*, as it did not detect hospitals performing significantly worse than average in 2011. Therefore, we propose to judge hospitals using the CM-V&O. This measure identified the hospitals with better outcomes the following year, and detected most of the hospitals performing significantly worse than average the following year, and was therewith most discriminative and reliable. Moreover, this simple measure is also understandable for all stakeholders. It may be argued that the CM-V&O, is too strict for the Dutch hospitals, as only 24% of all hospitals met the measure. By varying the ‘level of substandard care’ to for example an O/E mortality of 3 (e.g. 3 times higher mortality rate than expected based on the hospitals’ case-mix) the CM-V&O may be adjusted so that more hospitals meet the measure. However, as the number of hospitals in relation to the size of the Dutch population is exceptionally high, and therefore the average procedural volume low, it may be argued that the CM-V&O is not ‘too strict’, but that it exposes the limited accountability of Dutch hospitals in their current number. Possibly the CM-V&O is even more discriminative and reliable, and selects more hospitals when tested in a different, larger healthcare system.

Other studies have proposed similar composite measures using volume and outcome to assess hospital performances, [17] with similar results. However, they used the Empirical Bayes method to adjust outcomes for the procedural volume. The main difference between our approach and the Empirical Bayes method is that the Empirical Bayes method uses hospital rankings (from best to worst performing) instead of rating (better or worse than average), and therefore also takes into account the uncertainty of the position of the hospital, based on its performances, in relation to the position of the other hospitals. To identify outlier hospitals using the Empirical Bayes method, large procedural volumes are needed. Therefore, in the Dutch population, using the Empirical Bayes method, results in a ‘flat line’ with all hospitals performing ‘average’, and fails to identify better performing or underperforming hospitals. Moreover, a recent study describes that after the introduction of the DSCA not only national average outcomes improved, but also differences in hospital performance were reduced,[11] meaning that the position of one hospital, relative to another becomes more uncertain. This study proposes a more simple method, looking only at the position of the hospital in relation to the national average. Therewith, the CM-V&O does not classify the one hospital as performing better than the other, but simply classifies a hospital good enough or not good enough.

Although the CM-V&O performed better than the other two measures in identifying the better performing hospitals and detecting the underperforming hospitals, we found no significant difference in mortality in 2011 between hospitals that did and did not meet the CM-V&O in 2010. As Dishoeck et al. showed in their study, the ‘rankability’ of hospitals (the part of the heterogeneity between hospitals that is due to unexplained, hospital dependent differences) is highly dependent of the number of events in the different hospitals.[18] For mortality, the rankability is rather low meaning that most of the differences between hospitals is due to random variation and may thus be less suitable to rank hospitals. For morbidity on the other hand, rankability is much better as there is more systematic variation. This may also explain why the CM-V&O performed better for morbidity than for mortality. As mortality is just one aspect of quality, the CM-V&O should preferably be applied for both mortality and morbidity, or even for composite quality measures that combine both short and long term outcome, adverse as well as desirable.[19]

Our study resembles clinical audits or quality registration programs in other countries, such as the National Surgical Quality Improvement Program in the United States of America, or the various nation-wide registries in European countries. Some of these registrations also use the combination of volume and outcome to produce annual hospital ratings. However, they identify positive and negative outliers, but leave the majority of hospitals unclassified[20], [21], arguing that it cannot be proven that quality of care in these hospitals is insufficient. This line of reasoning differs from the relationship between providers and clients across many other areas of society, where the burden of proof for a good product lies with the provider, instead of the burden of proof for substandard quality lying with the client. The analogue in healthcare is that nowadays society will not settle for the lack of statistical proof that care is substandard, but demands evidence that the quality of care is adequate, in particular for high-risk procedures. The CM-V&O that we propose does exactly that.

Policy makers in many countries increasingly respond to societal concerns about health care safety and quality. In the Netherlands, societal demand for transparency has been formulated by the Dutch Health Care Inspectorate as the need for “justified trust”. Hospital volume has been chosen as a proxy for quality, backed up by enforced volume-based referral in an attempt to improve outcomes.[3], [22]. The present study suggests that CM-V&O is on both theoretical and practical grounds, better suited than the volume-measure to provide the “justified trust” in quality of care that society demands.
